# Exposure to fine particulate matter in the air alters placental structure and the renin-angiotensin system

**DOI:** 10.1371/journal.pone.0183314

**Published:** 2017-08-18

**Authors:** Sônia de Fátima Soto, Juliana Oliveira de Melo, Guilherme D’Aprile Marchesi, Karen Lucasechi Lopes, Mariana Matera Veras, Ivone Braga de Oliveira, Regiane Machado de Souza, Isac de Castro, Luzia Naôko Shinohara Furukawa, Paulo Hilário Nascimento Saldiva, Joel C. Heimann

**Affiliations:** 1 Department of Internal Medicine / Nephrology / Laboratory of Renal Physiopathology, University of São Paulo School of Medicine, São Paulo, SP, Brazil; 2 Department of Pathology / Pathology / Laboratory of Experimental Air Pollution, University of São Paulo School of Medicine, São Paulo, SP, Brazil; Faculty of Animal Sciences and Food Engineering, University of São Paulo, BRAZIL

## Abstract

Transforming growth factor beta 1 (TGFβ1), the uteroplacental renin-angiotensin system (RAS) and vascular endothelial growth factor A (VEGF-A) participate in the placentation process. The aim of this study was to investigate the effects of exposure to pollutants on the placenta.

**Methods:**

Female Wistar rats were exposed to filtered air (F) or to concentrated fine particulate matter (P) for 15 days. After mating, the rats were divided into four groups and again exposed to F or P (FF, FP, PF, PP) beginning on day 6 of pregnancy. At embryonic day 19, the placenta was collected. The placental structure, the protein and gene expression of TGFβ1, VEGF-A, and its receptor Flk-1 and RAS were evaluated by indirect ELISA and quantitative real-time PCR.

**Results:**

Exposure to P decreased the placental mass, size, and surface area as well as the TGFβ1, VEGF-A and Flk-1 content. In the maternal portion of the placenta, angiotensin II (AngII) and its receptors AT_1_ (AT_1_R) and AT_2_ (AT_2_R) were decreased in the PF and PP groups. In the fetal portion of the placenta, AngII in the FP, PF and PP groups and AT_2_R in the PF and PP groups were decreased, but AT_1_R was increased in the FP group. VEGF-A gene expression was lower in the PP group than in the FF group.

**Conclusions:**

Exposure to pollutants before and/or during pregnancy alters some characteristics of the placenta, indicating a possible impairment of trophoblast invasion and placental angiogenesis with possible consequences for the maternal-fetal interaction, such as a limitation of fetal nutrition and growth.

## Introduction

Fine atmospheric particulate matter (aerodynamic diameter between 2.5 and 10.0 μm—PM_2.5_) has the potential to cause adverse health effects [1,2]. The World Health Organization (WHO) has established a maximum exposure level of PM_2.5_ for humans as a daily mean of 25 μg/m^3^ [3].

Studies in humans showed that prenatal exposure to air pollutants influences fetal development [4] and increases the incidence of some diseases in postnatal life [5,6,7].

Exposure to air pollution during pregnancy causes disturbances in gestational development, fetal health [8] and low birth weight (LBW) [9] that are related to diseases in adulthood [10]. LBW and intrauterine growth restriction (IUGR) can occur due to placental structural and functional alterations [11,12] including an inappropriate maternal-fetal vascular interface, which is necessary to supply the bioenergetics needs of the fetus.

Studies from our laboratory concluded that in rats exposed to air pollution before and during pregnancy, the IL-4 content was elevated in fetal portion of the placenta. IL-4 is produced by Th2 cells, a cytokine present in pregnancy, but in the study, the increase was greater than concrol group. This result indicate a placental inflammatory reaction in response to fine particulate matter, suggesting a placental inflammatory response that may be one mechanism involved in deleterious effects during fetal development [12].

Van den Hooven and colleagues demonstrated that maternal exposure to elevated concentrations of PM_10_ is associated with low placental mass and lower proangiogenic (placental growth factor) and higher antiangiogenic factors (soluble fms-like tyrosine kinase 1) in the cord blood, which is consistent with an anti-angiogenic state [13].

The invasion of the maternal vasculature by the trophoblast is a prerequisite for the establishment of a normal placenta and the continuation of pregnancy. Several studies have suggested that transforming growth factor 1 (TGFβ1) has a role in the invasion of the endometrium [14]. In mammals, TGFβ1 can regulate a variety of cellular functions, including cell proliferation, differentiation, apoptosis and placental cell invasion [14,15,16].

Another factor that plays a role in placentation is vascular endothelial growth factor A (VEGF-A), which modulates angiogenesis by binding to its two receptors: fetal liver kinase 1 (Flk-1) and fms-related tyrosine kinase 1 (Flt-1). Flk-1 is a positive signal transducer, whereas Flt-1 is a suppressor of Flk-1 signaling [17,18,19,20].

Disorders of the uteroplacental renin angiotensin system (RAS) may lead to reduced uteroplacental blood flow [21,22]. In addition, local angiotensin II (AngII) is a potent regulator of trophoblast migration and invasion early in pregnancy [23].

In an epidemiological study from the Helsinki Birth Cohort, Barker and colleagues reviewed if measures of placental size could be used as markers of its function. They used the placental length and breadth to estimate the surface area, supposing that the surface area correlates with the exchange area and, consequently, the maternal-fetal interaction. They postulated that placental growth is polarized from the time of implantation, so that growth along the major axis, the length, is qualitatively different from growth along the minor axis. One possibility is that the placenta longitudinal diameter aligns with the fetal rostro-caudal growth, while the transverse diameter is an indicator of the transport of nutrients and oxygen from the mother to the fetus [24].

Exposure to air pollution is associated with a reduction in fetal weight in mice [25]. The fetal weight and growth is determined by the availability of nutrients to the fetus through the placenta [26]. Veras and colleagues described that ambient levels of particulate matter and other pollutants generated by urban traffic affects the maternal portion of the placenta and impairs fetal health [27].

Based on the studies that have found a relationship between an imbalance in RAS and disorders of pregnancy [28] and a relationship between exposure to air pollution and placental alterations, this study was designed to evaluate the effects of air pollution in terms of placental morphology and local RAS and the alterations of factors that influence the placentation process. In addition, another objective of this study was to evaluate if exposure to air pollutants before pregnancy may alter placental characteristics.

## Materials and methods

All experiments were approved by the Committee of Evaluation of Research Projects of the University of São Paulo School of Medicine in Brazil (certificate number 0381/10). All experiments are also compliant with the National Institutes of Health Guide for the Care and Use of Laboratory Animals (NIH Publications—eighth edition).

### Exposure protocol

To simulate real-life conditions where women are exposed to air pollution both before and during pregnancy, nine-week-old female rats were exposed to concentrated PM_2.5_ through a Harvard Ambient Particle Concentrator (HAPC) [29,30] 5 times per week for three weeks before pregnancy, resulting in 15 days of exposure, and/or seven times per week during pregnancy, resulting in 14 days of exposure. To avoid failure in blastocyst implantation, the exposure began on the 6^th^ day of pregnancy [31]. Mating was induced when the rats were 12 weeks old. When spermatozoids were found in the vaginal smear, this was considered the first day of pregnancy. The intention was to expose the animals daily to ambient air with a PM_2.5_ concentration of 600 μg/m^3^ for 1 hour in temperature- and humidity-controlled chambers. Each day, the initial ambient PM_2.5_ concentration was measured and the time of exposure was calculated. If the initial concentration was lower than 600 μg/m^3^, the exposure time was proportionally increased, and if the concentration was higher than 600 μg/m^3^, the exposure time was proportionally decreased. The limits for the exposure time were 30 to 90 minutes. The control animals were exposed to an identical daily exposure procedure, but they received filtered air instead. To monitor the amount of atmospheric particles to which the animals were exposed, a device called Data Ram was coupled to the pollution chamber.

The HAPC was located on the University of São Paulo School of Medicine campus, and the exposure protocols were conducted during the dry season from May to December 2011.

Between exposures, the animals were maintained in plastic cages (40x33 cm) at a controlled temperature in a 12-hour light-dark cycle; they also received clean air, food (CR-1 Nuvilab, Colombo, PR, Brazil) and water *ad libitum*.

### Animals

The four groups were evaluated according to exposure as follows: filtered air before and during pregnancy (the FF group); filtered air before pregnancy and polluted air during pregnancy (the FP group); polluted air before pregnancy and filtered air during pregnancy (the PF group); and polluted air before and during pregnancy (the PP group).

Body weight as well as food and water intake were measured weekly during the entire study protocol.

Tail-cuff blood pressure was measured weekly using the oscillometric method (model RTBP 2045 with the RTBP 001 acquisition system (Kent Scientific, Northwest Connecticut, USA) from the 9^th^ until the 14^th^ week of life, except during the 12^th^ week because of the mating period.

After the completion of each exposure period, the rats were fasted overnight, and the blood glucose level was determined using a glucometer (Advantage; Eli Lilly of Brazil Ltda, São Paulo, Brazil). Afterward, blood samples were collected, centrifuged and stored at -20°C until a radioimmunoassay was performed to detect the insulin levels (Rat Insulin RIA kit; LINCO Research Inc., St. Charles, MO, USA).

The overnight fasting glucose and insulin levels were used to calculate the HOMA (Homeostasis Model Assessment) index according to the following equation: (glucose (mmol/mL) x insulin (μUI/mL)/22.5).

On the 19^th^ day of pregnancy, the rats were anesthetized via an intraperitoneal injection of sodium pentobarbital (100 mg/kg). The abdominal wall was opened, the uterine vessels were ligated, and the uterus was carefully removed. The placentas were then dissected and weighed.

All placentas were easily separated by mechanically pulling into two regions. A region predominantly with maternal cells, called the maternal portion (mPl) and a region predominantly with fetal cells, called the fetal portion (fPl) and were stored in plastic tubes at -80°C.

Two dams per group were randomly selected, and the placental thickness, longitudinal diameter (LD) and transverse diameter (TD) were measured with a digital caliper. These diameters were determined according to the fetus position within the amniotic sac. The longitudinal diameter is the measure along the rostro-caudal axis, and the transverse diameter is perpendicular to the longitudinal diameter. These measures were used to calculate the surface area according to the following equation: (LD x TD x π)/4. The placentas of these two dams were not used to evaluate other parameters because of the excessive tissue manipulation and possible degradation.

### Maternal renin and angiotensin converting enzyme (ACE) activity

Plasma renin activity was determined using a radioimmunoassay kit (code CA-1533, Diasorin, Stillwater, Minnesota 55082–0285, USA). ACE activity was measured in the serum and in the placentas according to the method described by Santos *et al*. [32].

### Protein expression

The protein expression levels of TGFβ1, VEGF-A, Flk-1, Angiotensinogen, Renin, ACE, ACE2, AngII, AT_1_R, and AT_2_R were measured via an indirect ELISA according to the Proteimax Biotecnologia Ltda® protocol [33] described below.

Whole placentas from eight animals from each experimental group were homogenized (IKA ULTRA-TURRAX T10 Basic,Wilmington, North Carolina, USA) in 500 μL of buffer (50 mM Tris-Cl, 1 mM EDTA + 10% sucrose, pH 7.4), 5 μL of a cocktail of protease inhibitors (Sigma, St. Louis, Missouri, USA) and 100 mM sodium fluoride for each tissue. Homogenates were transferred to Eppendorf tubes and centrifuged at 15,000 × g for 10 minutes. Supernatants were discarded via tube inversion. The pellet was resuspended in 1000 μL of buffer (50 mM Tris-Cl, pH 7.4, 1 mM EDTA) and centrifuged at 15,000 × g for 40 minutes. The supernatants were discarded, and 500 μl buffer (50 mM Tris-Cl, pH 7.4) was added to the pellet and homogenized with a pipette. Protein concentration was measured using a kit (BCA protein assay, Thermo Fisher Scientific, Waltham, MA, USA). Equal amounts of protein (1 μg) were mixed with Tris buffer, pH 7.4, to complete a volume of 100 μl. Each sample was placed in a well of polystyrene ELISA plates (Costar 3590, 96-well flatbottom plate without a lid, high binding). The plate containing the samples was left in a clean room at room temperature for 24 hours to dry the samples via evaporation. The dried samples were blocked in blocking buffer (1% BSA, 5% sucrose, 0.05% sodium azide, PBS) for 180 minutes, incubated with primary antibodies of angiotensin II receptors AT1 and AT2. The plates were incubated at 4°C overnight. The plate was washed three times for five minutes with gentle agitation in PBS (200 μl per well) and incubated with 100 μl of a secondary antibody (anti-rabbit IgG, produced in goat and bound to alkaline phosphatase, SIGMA-ALDRICH, A3687, St. Louis, Missouri, USA,) for two hours under gentle agitation. Four washes were performed using PBS buffer for five minutes. After these washes, 100 μl of developing buffer was added (100 mM Tris-base, Sigma 104 phosphatase substrate) and read in an ELISA reader at a wavelength between 400 and 420 nm.

Placental tissue samples were weighed and homogenized in protein extraction buffer (RIPA lysis buffer, 100 mM phenylmethylsulfonyl fluoride, 10 mM sodium pyrophosphate, 100 mM sodium orthovanadate, protease inhibitor cocktail, and 100 mM sodium fluoride). The determination of the protein concentration was performed with an assay kit (Pierce BCA ® Protein Assay Kit; Thermo Scientific).

Equal amounts of protein were mixed in RIPA buffer at pH 7.4 to obtain a total volume of 100 μL, which was placed in polystyrene ELISA plates (Costar 3590–96 well—without lid, flat bottom, and high-binding). The plates were then dried for 24 hours at an ambient temperature.

After this procedure, the plates were treated with the following: PBS (100 mM sodium phosphate dibasic, sodium chloride 1370 mM, potassium chloride 27 mM, and potassium phosphate monobasic 20 mM) pH 7.4, treatment buffer (50 mM Tris-base) pH 7.4, formaldehyde 3.7%, and blocking buffer (1% BSA, 5% sucrose, and 0.05% sodium azide in 250 mL of PBS). They were then incubated with the appropriate primary antibody overnight at 4°C (Table 1).

**Table 1:** Antibodies used for ELISA protein expression protocol.

Antibody	Product code	Manufacturer, code and host
TGFβ1	SC-146	Anti- TGFβ1 C-146, Santa Cruz Biotechnology, Wembley, Middlesex, UK
VEGF-A	NB-100-2381	Anti-VEGF-A, Novus Biologicals Antibody, Colorado, USA
Flk-1	SC-504	Anti-Flk-1, Santa Cruz Biotechnology, Wembley, Middlesex, UK
AGTO	28101	Anti-Angiotensinogen, Immuno-Biological Laboratories, Minneapolis, USA
Renin	Bs-6184R	Anti-Renin, Bioss, Massachusetts, USA
ACE	SC20791	Anti-ACE H-170, Santa Cruz Biotechnology, Wembley, Middlesex, UK
ACE2	SC20998	Anti-ACE2 H-175, Santa Cruz Biotechnology, Wembley, Middlesex, UK
Ang II	GTX37789	Anti-Angiotensin II, Gene Tex International Corporation, Irvine, CA, USA
AT_1_R	SC1173	Anti-AT_1_ N-10, Santa Cruz Biotechnology, Wembley, Middlesex, UK
AT_2_R	SC9040	Anti-AT_2_ H-143, Santa Cruz Biotechnology, Wembley, Middlesex, UK

The host and isotype for all antibodies were rabbit and IgG.

On the following day, the plates were washed in PBS and incubated with a secondary antibody conjugated to alkaline phosphatase for 2 hours. Development buffer (100 mM Tris-base, Sigma 104 phosphatase substrate) was added, and then, the optic density was measured in an ELISA reader at a wavelength of 420 nm.

The results were expressed as percentage considering the FF group as 100%.

### Gene expression

Gene expression of TGFβ1, VEGF-A, Angiotensinogen, Renin, ACE, AT_1_aR and AT_2_R were evaluated by quantitative real-time PCR (qPCR) using total RNA extracted from the maternal and fetal portions of the placenta followed by reverse transcription to obtain cDNA. The qPCR was performed using the Rotor-Gene SYBR® Green RT-PCR Kit (Catalog no. 204074, Qiagen). The following primers were used (Table 2):

**Table 2:** Primers used for qPCR gene expression protocol.

Gene ID	Primer	Ampliconlength
Agt (NM_134432.2)	(Sense) 5'-CAC GGA CAG CAC CCT ATT T-3; (Anti-sense) 5'-GTT GTC CAC CCA GAA CTC AT-3'	100
ACE (NM_012544)	(Sense) 5'-AGA CTT GCC TGT GAC CTT TC-3'; (Anti-sense) 5'-CTG TGT AGA TGC TTG GGT GTA G-3'	102
Renin (NM_012642.4)	(Sense) 5'-GGA CAC TGG CAC ATC CTA TAT C-3'; (Anti-sense) 5'-ACC TGG CTA CAG TTC ACA AC-3'	114
AT_1_aR (NM_030985)	(Sense) 5'-TGT CAT GAT CCC TAC CCT CTA C-3'; (Anti-sense) 5'-GCC ACA GTC TTC AGC TTC AT-3'	105
AT_2_R (NM_012494)	(Sense) 5'-GCT GTG TTA ATC CCT TCC TGT A-3'; (Anti-sense) 5'-TAG TCT CTC TCT TGC CTT GGA-3'	108
TGFβ1 (AY550025)	(Sense) 5'–GCA ACA ATT CCT GGC GTT AC-3'; (Anti-sense) 5'-GTA TTC CGT CTC CTT GGT TCA G-3'	120
VEGF (AAY702972)	(Sense) 5'-CAA TGA TGA AGC CCT GGA GT-3'; (Anti-sense) 5'-TCT CCT ATG TGC TGG CTT TG-3'	96
GAPDH (NM_017008)	(Sense) 5'-GCA AGG ATA CTG AGA GCA AGA G-3'; (Anti-sense) 5'-GGA TGG AAT TGT GAG GGA GAT G-3'	98

### Statistical analysis

Data are presented as the mean±standard error of the mean (SEM). Differences were evaluated using ANOVA with Student–Newman–Keuls or Tukey post hoc test. P<0.05 was considered statistically significant. Statistical analysis was performed using version 5 of GraphPad Prism (GraphPad®, San Diego, CA, USA).

## Results

The detailed results can be accessed in the file that appears in Supporting Information (S1 Dataset).

### Maternal parameters

Body weight, food and water intake, blood pressure, blood glucose, serum insulin and HOMA index were not influenced by exposure to particulate matter before and/or during pregnancy. However, all these parameters were influenced by pregnancy in all experimental groups (Table 3).

**Table 3:** Maternal parameters.

	FF	FP	PF	PP
Body weight (g)				
BP	236.2±5.0 (9)	234.6±5.8 (11)	231.0±4.8 (9)	240.6±6.4 (8)
DP	312.7±7.8 (9)*	313.0±9.0 (11)*	304.9±8.7 (9)*	313.7±9.4 (8)*
Food intake (g/week)				
BP	143.4±2.3 (9)	148.5±3.65 (11)	130.4±2.28 (8)^a^^,^^b^	130.9±2.57 (8)^a^^,^^b^
DP	199.6±12.00 (8)*	198.7±11.44 (9)*	186.0±10.10 (9)*	189.9±8.99 (6)*
Water intake (mL/week)				
BP	216.2±301 (9)	219.9±6.26 (10)	217.6±6.29 (9)	194.2±6.50 (8)^a^^,^^b^^,^^c^
DP	332.5±17.7 (7)*	317.9±19.45 (10)*	305.0±15.72 (9)*	302.5±17.97 (6)*
Blood pressure (mmHg)				
BP	112.6±1.4 (6)	113.4±1. (7)	116.9±1.0 (7)	115.6±1.6 (7)
DP	115.1±2.2 (6)	115.9±1.6 (7)	114.8±2.0 (7)	117.2±1.9 (7)
Blood glucose (mmol/L)				
BP	102.7±5.4 (7)	98.6±2.1 (9)	98.8±3.4 (8)	105.4±2.1 (8)
DP	88.4±2.8 (7)*	88.2±2.3 (9)*	85.5±2.5 (8)*	89.3±3.2 (8)*
Serum insulin (ng/mL)				
BP	0.36±0.1 (5)	0.35±0.1 (7)	0.40±0.1 (5)	0.36±0.1 (3)
DP	0.93±0.3 (5)*	0.72±0.3 (6)*	0.85±0.2 (6)*	0.64±0.2 (6)*
HOMA index (mmols.mU.l^-1^)				
BP	2.2±0.4 (5)	2.0±0.5 (7)	2.2±0.3 (5)	2.2±0.7 (3)
DP	5.0±1.5 (6)*	3.7±1.6 (6)*	4.0±0.9 (6)*	3.1±0.9 (6)*

BP: before pregnancy. DP: during pregnancy. FF: exposed to filtered air before and during pregnancy; FP: exposed to filtered air before pregnancy and polluted air during pregnancy; PF: exposed to polluted air before pregnancy and filtered air during pregnancy; and PP: exposed to polluted air before and during pregnancy; (n): number of samples per group. Results are shown as the mean±SEM.

*p<0.05 vs BP in the same corresponding group. p<0.05

^a^vs FF

^b^vs FP

^c^vs PF.

### Placental structure

**Fig 1:**
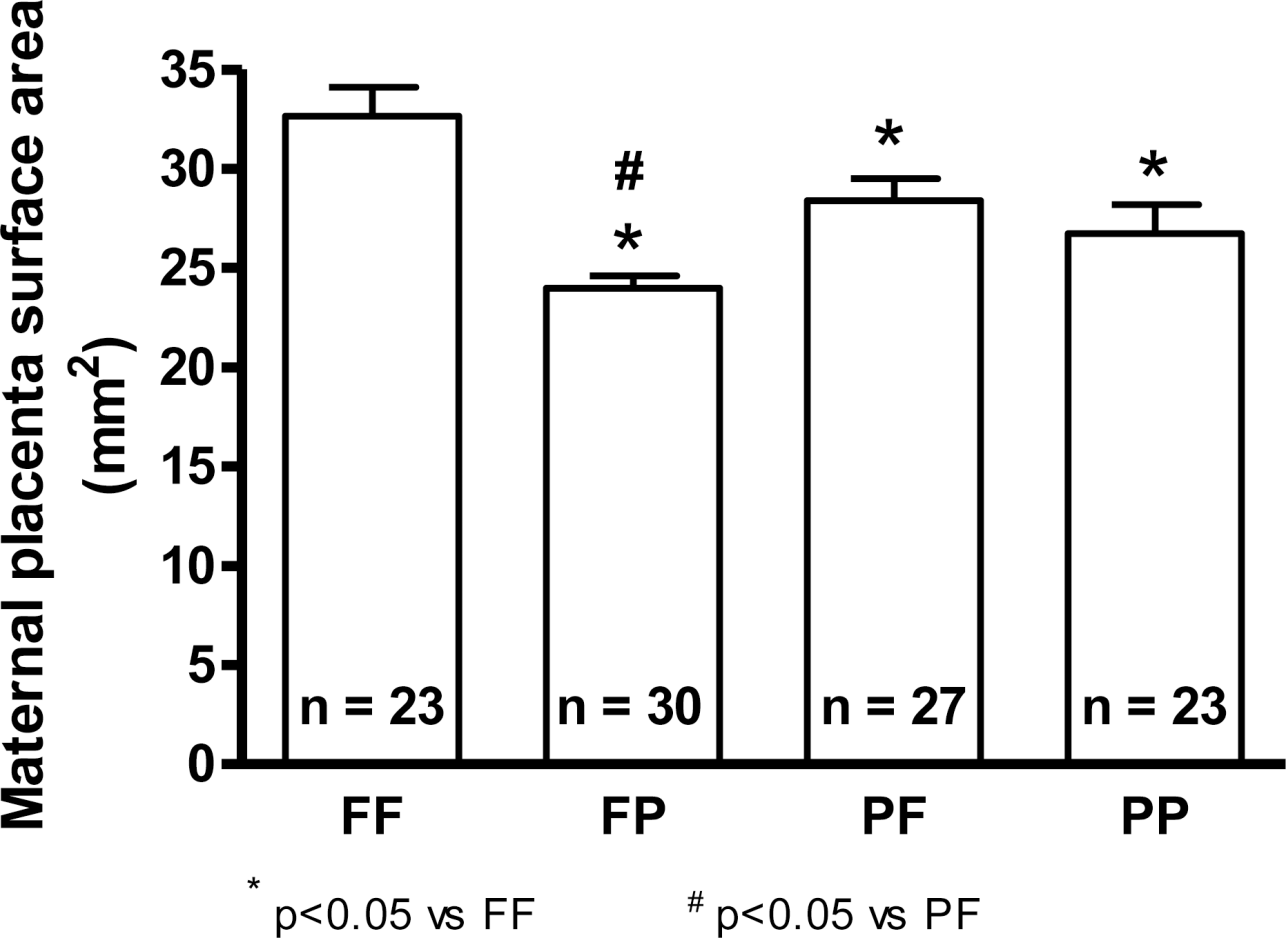
Surface area of the maternal placenta. FF = filtrated air before and during pregnancy, FP = filtrated air before and polluted air during pregnancy, PF = polluted air before and filtrated air during pregnancy, PP = polluted air before and during pregnancy. *p<0.05 vs. FF. #p<0.05vs. PF.

Evaluation of the placental structure in response to exposure to pollution indicates that all parameters were negatively influenced when exposure was performed mainly before gestation. An exception was observed on the effect on the placental longitudinal diameter. Interestingly, the placental surface area, an indicator of maternal fetal nutrient transfer, was lower when exposure to particulate matter occurred before and/or during pregnancy (Fig 1 and Table 4).

**Table 4:** Mass, thickness, longitudinal and transversal diameters and surface area of the placenta.

	FF	FP	PF	PP
Mass (g)				
mPl	0.14±0.006 (93)	0.15±0.008 (128)	0.12±0.006 (115) ^a^^,^^b^	0.12±0.008 (108) ^a^^,^^c^
fPl	0.43±0.006 (99)	0.44±0.007 (134)	0.41±0.005 (121)^a^^,^^b^^,^^d^	0.43±0.004 (113)
Thickness (mm)				
mPl	3.15±0.1 (23)	2.78±0.1 (30)^a^	2.92±0.1 (26)	2.96±0.1 (23)
fPl	4.42±0.1 (24)	4.17±0.1 (33)	3.93±0.1 (27)^a^	4.40±0.1 (23)
LD (mm)				
mPl	6.64±0.2 (23)	5.94±0.1 (30)^a^^,^^d^	6.29±0.1 (26)	6.45±0.2 (23)
Pl	12.72±0.2 (24)	11.83±0.2 (33)^a^	11.90±0.2 (27)^a^	12.31±0.2 (23)
TD (mm)				
mPl	6.21±0.1 (23)	5.15±0.1 (30)^a^^,^^c^	5.69±0.1 (26)^a^	5.21±0.2 (23)^a^^,^^c^
fPl	11.76±0.2 (24)	9.95±0. (33)^a^^,^^c^^,^^d^	10.79±0.2 (27)^a^	10.52±0.1 (23)^a^
Surface area (mm^2^)				
mPl	32.64±1.46 (23)	24.0±0.6 (30)^a^^,^^c^	28.4±1.1 (27)^a^	26.73±1.5 (23)^a^
fPl	117.6±2.4 (24)	90.2±2.2 (33)^a^^,^^c^^,^^d^	101.1±3.0 (27)^a^	101.7±2.3 (23)^a^

mPl: maternal portion of the placenta. fPl: fetal portion of the placenta. LD: longitudinal diameter; TD: transversal diameter; FF: exposed to filtered air before and during pregnancy; FP: exposed to filtered air before pregnancy and polluted air during pregnancy; PF: exposed to polluted air before pregnancy and filtered air during pregnancy; and PP: exposed to polluted air before and during pregnancy; (n): number of samples per group. Results are shown as the mean±SEM. p<0.05

^a^vs FF

^b^vs FP

^c^vs PF

^d^vs PP.

### Protein expression of the RAS components

Exposure to particulate matter on AT1R, AT2R, AngII and renin protein expression was different among fPl and mPl. Angiotensinogen expression did not differ between the groups in the mPl or the fPl. ACE expression in both the mPl and the fPl was lower in the PF and PP groups than in the FF and FP groups (Table 5).

**Table 5:** Protein expression of AGTO, renin, ACE, ACE2, AT1R and AT2R receptors, angiotensin II and ACE and plasma renin activities in the maternal and fetal portions of the placenta.

	FF	FP	PF	PP
Protein expression (%)				
AGTO				
mPl	100.0±0.0 (7)	104.9±10.8(7)	114.6 ±19.3 (6)	85.9±4.3 (6)
fPl	100.0±0.0 (9)	121.7±15.7 (8)	127.9±10.3 (7)	115.3±11.4 (7)
Renin				
mPl	100.00±0.0 (7)	139.0±11.1 (6)	113.5±18.6 (5)	120.7±26.4 (8)
fPl	100.0±0.0 (5)	192.8±27.8 (7)^a^	204.3±21.4 (7)^a^	149.9±20.0 (7)^a^
ACE				
mPl	100.0±0.0 (7)	97.0±4.4 (8)	82.0±3.1 (8)^a^^,^^b^	80.4±4.9(8)^a^^,^^b^
fPl	100.0±0.0 (8)	90.2±5.3 (8)	74.2±4.8 (8)^a^^,^^b^	59.8±3.4 (8)^a^^,^^b^
ACE2				
mPl	100.0±0.0 (7)	107.6±11.4 (8)	84.3±5.6 (8)	71.8±5.6 (8)^a^^,^^b^
fPl	100.0±0.0 (7)	110.8±5.8 (8)	97.0±7.7 (8)	87.5±3.7 (8)^b^
AT1R				
mPl	100.0±0.0 (7)	99.7±11.19 (8)	75.6±5.2 (7)	45.8±4.02 (8)^a^^,^^b^^,^^c^
fPl	100.0±0.0 (7)	202.1±41.16 (7)^a^	118.7±17.69 (8)^b^	142.3±15.91 (5)
AT2R				
mPl	100.0±0.0 (7)	91.1±8.0 (8)	73.9±10.4 (8)	39.8±7.1 (8)^a^^,^^b^^,^^c^
fPl	100.0±0.0 (7)	97.1±8.5 (7)	63.7±11.22 (8)^a^^,^^b^	39.5±7.5 (6)^a^^,^^b^
Angiotensin II				
mPl	100.0±0.0 (7)	104.5±11.72 (6)	105.2±8.8 (5)	76.0±3.3 (8)^a^^,^^b^^,^^c^
fPl	100±0.0 (8)	90.5±3.71 (8)^a^	90.8±2.3 (8)^a^	83.28±2.1 (8)^a^
ACE activity				
Serum (nmol His-Leu.min^-1^.mL^-1^)	33.50±1.39 (9)	28.89±1.37 (9)^a^	28.90±1.67 (7)^a^	26.53±1.06 (8)^a^
mPl (nmol His-Leu.min^-1^.mg^-1^)	0.8±0.07 (5)	0.7±0.06 (9)	0.6±0.05 (8)^a^	0.5±0.05 (6)^a^
fPl (nmol His-Leu.min^-1^.mg^-1^)	1.1±0.07 (6)	1.0±0.11 (6)	1.1±0.10 (7)	1.4±0.24 (6)
Plasma renin activity (ng.mL^-1^.hr^-1^)	24.82±1.84 (7)^b^^,^^c^	30.54±1.40(8)	31.97±0.80 (7)	21.69±3.11 (7)^b^^,^^c^

mPl: maternal portion of placenta. fPl: fetal portion of placenta. AGTO: angiotensinogen. ACE: angiotensin I converting enzyme. ACE2: angiotensin I converting enzyme type 2. AT1R and AT2R: angiotensin II receptor type 1 and type 2. TGFβ1: transforming growth factor beta 1. FF: exposed to filtered air before and during pregnancy; FP: exposed to filtered air before pregnancy and polluted air during pregnancy; PF: exposed to polluted air before pregnancy and filtered air during pregnancy; and PP: exposed to polluted air before and during pregnancy; (n): number of samples per group.Results are shown as the mean±SEM. p<0.05

^a^vs FF

^b^vs FP

^c^vs PF.

### Serum and placental ACE activity and plasma renin activity

Serum ACE activity was lower in all groups that were exposed to air pollution either before and/or during pregnancy. Similar to ACE protein expression, ACE activity in the mPl was lower in the PF and PP groups than in the FF group. ACE activity in the fPl did not differ among the experimental groups (Table 3).

Plasma renin activity at the end of pregnancy was higher in the FP and PF groups than in the FF and PP groups (Table 5).

### TGFβ1 protein expression

TGFβ1 protein expression in the mPl was lower in the FP, PF and PP groups than in the FF group and was even lower in the PF and PP groups than the FP group. In the fPl, TGFβ1 protein expression was lower in the FP and PF groups than in the FF group. (Table 6).

**Table 6:** Protein expression of VEGF-A, Flk-1 and TGFβ1 in the maternal and fetal portions of the placenta.

Protein expression (%)	FF	FP	PF	PP
VEGF-A				
mPl	100±0 (7)	85.3±2.6 (6)^a^	87.7±3.5 (7)^a^	77.4±1.3 (6)^a^^,^^b^^,^^c^
fPl	100±0 (7)	86.7±2.8 (8)^a^	90.0±4.5 (8)	82.9±4.1 (8)^a^
Flk-1 receptor				
mPl	100±0 (7)	89.9±3.7 (6)	94.9±5.7 (8)	77.4±5.0 (6)^a^^,^^c^
fPl	100±0 (7)	82.2±5.2 (8)^a^	56.2±6.3 (8)^a^^,^^b^	45.3±6.8 (7)^a^^,^^b^
TGFβ1				
mPl	100.0±0.0 (7)	72.7±6.4 (8)^a^	48.6±5.9 (8)^a^^,^^b^	49.5±5.8 (8)^a^^,^^b^
fPl	100.0±0.0 (7)	76.9±8.6 (8)^a^	76.6±3.8 (8)^a^	83.3±6.0 (8)

mPl: maternal portion of the placenta. fPl: fetal portion of the placenta. (n): samples per group. FF: exposed to filtered air before and during pregnancy; FP: exposed to filtered air before pregnancy and polluted air during pregnancy; PF: exposed to polluted air before pregnancy and filtered air during pregnancy; and PP: exposed to polluted air before and during pregnancy; (n): number of samples per group.Results are shown as the mean±SEM. p<0.05

^a^vs FF

^b^vs FP

^c^vs PF.

### VEGF-A and Flk-1 receptor protein expression

Compared to the FF group, VEGF-A protein expression in the mPl was lower in all groups that were exposed to PM_2.5,_ and it was even lower in the PP group compared with the FP and PF groups. Flk-1 expression was lower in the PP group than in the FF and PF groups. In the fPl, VEGF-A expression was lower in the groups that were exposed to PM_2.5_ during pregnancy, whereas Flk-1 was lower in the FP, PF and PP groups compared with the FF group and was also even lower in the PF and PP groups compared with the FP group. (Table 6).

### Gene expression

Gene expression of TGFβ1, angiotensinogen, ACE, renin, AT1aR and AT2R were not different among experimental groups. However, VEGF-A gene expression was lower in the PP (1.311±0.058, n = 8) group than in the FF (1.706±0.174, n = 7) group in the fPl.

### Fetal parameters

Exposure to particulate matter before and/or during pregnancy did not change the litter size and body weight (Table 7).

**Table 7:** Fetal parameters on the 19^th^ day of pregnancy.

	FF	FP	PF	PP
Litter size (number of fetuses)	15.0±0.4 (9)	15.3±0.7 (10)	15.4±0.5 (9)	14.5±0.7 (8)
Litter body weight (g)	24.2±1.1 (9)	25.6±1.7 (10)	25.0±1.2 (9)	24.1±1.1 (8)

mPl: maternal portion of the placenta. fPl: fetal portion of the placenta. (n): samples per group. FF: exposed to filtered air before and during pregnancy; FP: exposed to filtered air before pregnancy and polluted air during pregnancy; PF: exposed to polluted air before pregnancy and filtered air during pregnancy; and PP: exposed to polluted air before and during pregnancy; (n): number of samples per group.Results are shown as the mean±SEM. p<0.05

^a^vs FF

^b^vs FP

^c^vs PF.

## Discussion

Air pollution has been associated with many adverse health outcomes including alterations during pregnancy [34,35]. However, in the present study, exposure to particulate matter before and/or during pregnancy did not alter hormonal and metabolic changes observed during normal gestation such as body weight gain [36,37], increased food intake [38], insulin resistance [39], etc. The higher body weight and food intake during pregnancy indicate that undernutrition is probably not a mechanism stimulated by exposure to PM_2.5_ before and/or during pregnancy. Exposure to filtered air during pregnancy did not reverse the lower placental mass of both the maternal and fetal portion in rats exposed to PM_2.5_ before pregnancy, indicating that the effect of air pollution is irreversible despite the correction of environmental conditions during pregnancy.Interestingly, if the exposure to PM_2.5_ persists during pregnancy, the placental mass of the fetal portion is not lower than that in the PF group. Further studies should be done to elucidate the mechanisms underlying this finding. However, it is worthwhile to verify if persistent exposure to air pollution stimulates some protective mechanisms such as an increase of a growth factor or the inhibition of a growth factor inhibitor.

Based on the findings of the circulating renin and ACE activities, it can be conjectured that increased plasma renin activity enhances angiotensin I expression. Since it is known that enzyme activity is stimulated by an increased amount of substrate, the finding of lower serum ACE activity is possibly due to a lower circulating concentration of this enzyme. Serum ACE is mainly produced in the pulmonary circulation, which is altered in response to PM_2.5_ exposure [38].

Placental AngII is an important factor that regulates trophoblast invasion and migration [23]. In the present study, placental AngII was lower in response to the exposure to particulate matter, which suggests a possible decrease in trophoblast invasion and maternal-fetal interaction. The longitudinal diameter in the FP and PF groups and the transverse diameter of the placenta in all experimental groups were decreased in response to the exposure to pollutants. Therefore, the placental surface area was decreased, which suggests an impairment in the maternal-fetal interaction because the surface area of the placenta is an indicator of this function [24].

We have shown that AT_1_R protein expression is higher in the fPl of the FP group, which indicates that exposure to PM_2.5_ may activate this receptor in the fPl and that this may predispose the fPl to a decrease in trophoblast invasion [40,41] and IUGR.

Lower AT_2_R protein expression in the placenta may also compromise the uteroplacental blood flow [40,41].

Different from the findings of placental mass determinations, the placenta size parameters are lower in the FP group both in the maternal and fetal portions. Lower size along with unchanged mass is indicative of a higher density, which can be due to decreased water or increased solid content. Increased solid content may be due to inflammation as found in a previous study from our laboratory [12]. A possible explanation for the effect of PM_2.5_ exposure on the placental mass is that dams exposed before pregnancy may already be contaminated by particulate matter in the initial steps of pregnancy, and this may impair placental development. In the FP group, exposure occurred after placental implantation and could explain the absence of an alteration in the placental mass in this group. The fetal portion of the placenta was not influenced by exposure to PM_2.5_ in the PP group, which may be due to a compensatory growth of this portion due to a lower mass of the maternal portion of the placenta in this group.

Exposure to air pollution in early pregnancy may cause insufficient trophoblast formation; this may lead to impaired placental vascularization, which results in fetal hypoxia and IUGR [18]. Studies also suggest that TGFβ1 may play a crucial role in the invasion of trophoblast cells [14] and the stimulation of morphological, molecular and functional differentiation of the mouse trophoblast *in vitro* [42]. Lower placental TGFβ1 expression in the groups that were exposed to air pollution may indicate impairment in the differentiation and invasion of trophoblasts.

According to Holmes and Zachary (2005), VEGF-A and its receptors have key biological roles in the formation of vascular structures. The lower placental VEGF-A and Flk-1 concentrations in the groups that were exposed to polluted air suggest deficient vascularization of the placental tissue with possible alterations in the exchange of nutrients and gases between the mother and fetus, which would compromise its development [20].

Regarding the gene expression of the RAS components TGFβ1 and VEGF-A, the discrepancies between the results in relation to protein expression may be due to possible changes in the synthesis or degradation of these proteins. Additional studies are needed to better understand these results.

This possibility offers a new and interesting clue for future investigation that may add new information for further understanding the long-term influence of prenatal conditions on diseases with fetal origins.

The findings regarding exposure to particulate matter before pregnancy need further studies for its full comprehension. However, a speculation based on the study of Li et al. (2005) is that alteration in tissue RAS induced by exposure to particulate matter before pregnancy may influence fetal and/or placenta development [43]. The absence of changes in fetal size on day 19 of pregnancy does not exclude alterations in fetal growth since very rapid fetal growth occurs in the last two days of pregnancy.

## Conclusions

Finally, the findings of the present study indicate that exposure to PM_2.5_, even within the limits established for safety by the WHO, may potentially alter placental RAS and compromise the maternal-fetal interaction, which could impair fetal nutrition and growth. The absence of histological evidences and immunolocalization is a limitation of the present study. This limitation will be corrected in the next study of this experimental line.

## Supporting information

S1 Dataset Results.pzf. (PZF)
